# An Assessment of Motor Skills in Infants at Risk of Atypical Psychomotor Development Using the Vojta Method

**DOI:** 10.3390/children12080976

**Published:** 2025-07-24

**Authors:** Robert Podstawski, Katarzyna Balewska-Juras, Krzysztof Borysławski, Attila Szabo, Jadwiga Snarska

**Affiliations:** 1 Human Wellness Research Laboratory, Department of Physiotherapy, School of Public Health, University of Warmia and Mazury in Olsztyn, 10-957 Olsztyn, Poland; kbalewska.juras@wp.pl; 2 Institute of Health, Angelus Silesius University of Applied Sciences in Wałbrzych, 58-300 Wałbrzych, Poland; kboryslawski@ans.edu.pl; 3 Faculty of Health and Sport Sciences, Széchenyi István University, 9026 Győr, Hungary; szabo.attila@sze.hu; 4 Department of General Surgery, School of Public Health, University of Warmia and Mazury in Olsztyn, 10-957 Olsztyn, Poland; jadwiga.snarska@uwm.edu.pl

**Keywords:** physical therapy diagnosis, motor disorders, coordination of the central nervous system, postural reflexes, spontaneous motor activity

## Abstract

**Background**: Some neonates are assessed for the risk of atypical psychomotor development at birth and are referred for reflex locomotion therapy using the Vojta method. **Aim**: The aim of this study was to analyze the relationships between spontaneous motor activity (SMA), ideal movement patterns (IMPs), central coordination disorders (CCDs), vital signs at birth, involuntary reflexes, and postural asymmetry in infants. **Methods**: This study involved 90 female and 107 male subjects in the age interval of 1–16 months (4.15 ± 2.18). Their psychomotor development was assessed using the Vojta method. Age-appropriate involuntary reflexes were evaluated, and both parameters were correlated with perinatal risk factors. **Results**: Males scored significantly higher than females (difference of −0.7, *p* = 0.022) in the SMA test. In both genders, SMA (*p* < 0.001 in both genders) and IMP scores improved significantly with age. In male infants, higher CCD scores were associated with significantly lower SMA and IMP scores (*p* = 0.017 and *p* < 0.001, respectively). Significantly higher CCD scores were noted in female subjects with the Moro reflex and postural asymmetry (*p* = 0.003 and *p* = 0.002, respectively). In males, the Moro reflex was significantly correlated with the Vojta reaction (*p* = 0.012) and the Collis vertical suspension reflex (*p* < 0.001). **Conclusions**: Vital signs at birth, including birth weight, Apgar score, and type of delivery, can predict motor development disorders but do not clearly differentiate infants that require neurodevelopmental therapy.

## 1. Introduction

In physical therapy practice, an accurate diagnosis is essential for initiating effective treatment in all age groups. Any deviations or delays in the psychomotor development of infants and young children should be immediately addressed to initiate early therapeutic interventions and prevent psychomotor disorders in later life [1]. However, children younger than 12 months are challenging to diagnose because their central nervous system is not yet fully developed [2,3]. In some cases, delayed myelination of nerve fibers can disrupt neural and muscular coordination, and these symptoms can be misdiagnosed [4]. For this reason, infants with prenatal or perinatal risk factors should receive specialized care immediately after birth and should be subjected to a full range of diagnostic tests.

Early psychomotor development and disorders of the central nervous system (CNS) are diagnosed with the use of advanced imaging methods, including magnetic resonance imaging (MRI), transfontanellar ultrasound (TFUS), and electroencephalography (EEG) [5,6]. These imaging techniques enable early detection of developmental defects and neonatal encephalopathy, facilitating quick initiation of therapeutic interventions [7]. In neonates, physical therapists also assess primitive reflexes, gross motor skills, positional preferences, and spontaneous movements and posture [8,9]. The most popular diagnostic methods include Vojta therapy [10,11], the Bobath concept (neurodevelopmental treatment, NDT-Bobath) [12,13,14], Prechtl’s method [15,16], and the Munich Functional Developmental Diagnostics (MFDR) scale [17,18].

Vojta therapy is a widely used diagnostic tool in clinical practice that relies on developmental kinesiology and reflex locomotion principles. The Vojta method can be used to diagnose and treat neurophysiological disorders from birth to the achievement of bipedal locomotion [19,20]. This approach examines spontaneous movement patterns, automatic involuntary motor reactions, and seven postural reactions in young children. The development of the CNS influences these reactions and movement patterns. In Vojta therapy, special attention is given to spontaneous motor activity (SMA), postural reactions, the level of maturity of the central nervous system, and, above all, ideal movement patterns (IMPs) [21,22]. In this approach, other reflexes involved in postural control and SMA are also evaluated based on observations of the child’s primitive reflexes and automatic reactions, including head control, the uprighting and anti-gravity mechanisms of different body segments, and the ability to prop up on the hands or elbows for support in a prone or sitting position. These milestones follow a predictable sequence in successive neurodevelopmental stages, with contextual variations in the external output that guide the diagnosis [23]. The diagnosis provides information about non-retained disorders of the nervous system that can be effectively resolved through physical therapy involving neurophysiological methods [24].

Atypical psychomotor development of infants exposed to various risk factors during the prenatal period, delivery, and the perinatal period has been explored in numerous studies. Research has shown that perinatal risk factors can lead to neurological disorders and can be captured by the deviations in the output observed in a neurophysiological test conducted using the Vojta method [22,23]. However, the relationships between central coordination disorders (CCDs) in infants diagnosed with the Vojta method and perinatal risk factors such as gestation length, type of delivery, birth weight, Apgar score, and primitive reflexes have never been found in the literature. These links should be identified to facilitate the early detection of psychomotor anomalies in infants and to assist neonatologists and pediatricians in implementing therapeutic approaches that can significantly contribute to the healthy development of children.

Therefore, this study aimed to identify the relationships between the psychomotor skills of infants assessed with the Vojta method based on their SMA, IMP, and CCD scores and perinatal risk factors, involuntary reflexes, and postural asymmetry.

## 2. Materials and Methods

### 2.1. Participants

This study involved 197 infants at risk of atypical psychomotor development who were patients of the Rehabilitation Clinic of the Center for Rehabilitation and Education in Szczytno and were referred for Vojta physiotherapy by a physician (a rehabilitation consultant) to confirm or rule out developmental disorders. The study group consisted of 107 male subjects aged 4.15 ± 2.18 months and 90 females aged 3.84 ± 2.12 months (1–16 months). The inclusion criteria were complications during pregnancy and delivery, preterm birth, a low Apgar score in the first minutes after birth, a low birth weight, postural asymmetry (at 7 weeks of age), atypical muscle tone, delayed psychomotor development, and non-age-appropriate involuntary reflexes. During the study, all children were healthy and did not present with any symptoms of infection. Children with genetic disorders were excluded from the study.

### 2.2. Ethical Statement

This study was conducted in accordance with the guidelines and policies of the Health Science Council and the Declaration of Helsinki. The study received approval from the Ethics Committee of the Medical University of Poznań (179/2012). Each child’s parent was provided with detailed information about the study’s purpose, potential risks, procedures, and relevant matters. All parents gave voluntary informed consent to participate in the study and agreed to the publication of anonymous data.

### 2.3. Procedures, Data Collection, and Equipment

The psychomotor development of the studied infants, including potential disorders, was assessed. Before the tests, information regarding the progression of pregnancy, maternal diseases, gestation length, type of delivery (spontaneous or with surgical intervention), the child’s birth weight, Apgar score, and adaptation to life in the first days after birth was collected from the parents.

Spontaneous motor activity was evaluated in both supine and prone positions, based on the number of presented skills. Ideal movement patterns were evaluated in the supine position during rolling, sitting, toddling, standing up, and walking. Special attention was placed on postural asymmetry. Any deviations in the alignment of different body parts relative to the midline axis were evaluated in supine and prone positions. Postural asymmetry was diagnosed based on the tilt of the head towards the shoulder girdle (always the same shoulder), obliquity of the shoulder and pelvic girdles, and clear preference for holding the head rotated or the body always towards one side only. The same criteria were applied to diagnose postural asymmetry in sitting and standing positions. In the next stage, primitive reflexes were assessed considering the child’s age and CNS development. The test involved evaluating spinal and tonic reflexes, as well as assessing the ability to support the head and control head movements, maintain an upright position and balance, and prop up on the hands or elbows for support in both prone and sitting positions. The child’s reactions to and specific behaviors in each test were registered.

#### Assessment Involving the Vojta Method

The rate of motor development was assessed using the seven postural reactions proposed by Vojta for neuromotor screening [25]. In healthy children, these reactions correspond to typical stages of ontogenic development of postural and locomotive skills during the first year of life [11]. These skills evolve with age and follow a predictable sequence in successive stages of psychomotor development [25]. Atypical reactions that deviate from the pattern described by Vojta are referred to as CCDs. The severity of CCDs is evaluated on a four-point scale based on the number of atypical postural reactions:

1. Very mild—1–3 atypical postural reactions;
2. Mild—4–5 atypical postural reactions;
3. Moderate—6–7 atypical postural reactions;
4. Severe—7 atypical postural reactions [26,27].

The infants’ involuntary and automatic reactions to changes in body position were evaluated during the test, and all children received a CCD score based on the above rating scale. Support and extension postures, changes in muscle tone, alignment of the spine and enarthroses, and stages of postural and movement output were assessed in the postural reaction test.

The assessments were conducted by a certified Vojta therapist in the presence of the parents. Each test was conducted under identical, optimal conditions at constant temperature and in a quiet room. Most of the examined infants participated in neurodevelopmental therapy after the study. The children were evaluated for the achievement of age-specific developmental milestones (the age of pre-term infants was adjusted accordingly) and for the presence of typical or atypical postural reactions. The motor skills and CCD scores of male and female participants were compared separately. The presence of significant relationships between vital signs at birth, SMA, IMPs, the severity of CCDs, involuntary reflexes, and postural asymmetry was determined.

### 2.4. Statistical Analysis

The significance of the differences between means was determined by Student’s *t*-test for independent variables. The relationships between quantitative traits were analyzed by calculating Pearson correlation coefficients (r). The chi-square test was used to analyze the relationships between nominal (category) features. The gamma (γ) coefficient numerically equivalent to Pearson’s r was also calculated. The results were processed statistically in Statistica PL v. 13.0 at a significance level of *p* < 0.05.

## 3. Results

The examined infants were born between 24 and 42 weeks of pregnancy. On average, male subjects were born at 37.5 ± 3.5 weeks, and females were born at 38.4 ± 2.7 weeks. Birth weights were similar in both genders, with an average of 3118.5 ± 859.2 g in males and 3078.4 ± 783.2 g in females. Neonates were evaluated on the 10-point Apgar test in the first minute after birth. The average score was 8.9 ± 2.0 in males and 9.3 ± 1.4 in females. The study population is characterized in Table 1.

Male subjects received significantly higher scores than females (difference of −0.7, *p* = 0.022) only in the assessment of SMA. No significant differences in CCD or IMP scores were observed between the genders (*p* = 0.151 and *p* = 0.321, respectively) (Table 2).

In both genders, SMA (*p* < 0.001 in both genders) and IMP (*p* < 0.001 in both genders) scores improved significantly with age. In female subjects, a higher birth weight and higher Apgar scores were associated with significantly higher SMA and IMP scores (*p* < 0.05) (Table 3). In both genders, gestation length was not significantly correlated with SMA or IMP scores.

In the study population, CCD scores were not significantly correlated with vital signs at birth or the infant’s age on the day of the assessment (Table 4).

The relationships between CCD, SMA, and IMP scores are presented in Table 5. Significant correlations were found only in male subjects. In males, a significant inverse correlation was noted between the CCD score and the SMA and IMP scores (*p* = 0.017 and *p* < 0.001, respectively).

Potential relationships between the number of atypical postural reactions (CCD score) and the type of delivery (spontaneous or with surgical intervention), involuntary motor reflexes, and postural asymmetry were determined in a detailed analysis (Table 6). Female infants with the Moro reflex and postural asymmetry received significantly higher CCD scores (*p* = 0.003 and *p* = 0.002, respectively). The number of atypical postural reactions (CCD score) was also significantly higher in males with the palmar grasp reflex and postural asymmetry (*p* = 0.049 and *p* < 0.001, respectively). In the group of male and female infants born by cesarean section, CCD scores were significantly higher in females (*p* = 0.018).

The results of the analysis examining the relationships between the Moro reflex and seven postural reactions (CCD score) are presented in Table 7. In males, the presence of the Moro reflex was significantly correlated only with the Vojta reaction (*p* = 0.012) and the Collis vertical suspension response (*p* <0.001). In male infants, the relationship between the Moro reflex and the Landau reaction was on the borderline of statistical significance (*p* = 0.053). In female subjects, postural reactions (CCD score) were not significantly correlated with the Moro reflex (*p* > 0.05). Only the correlation between the Moro reflex and the pull-to-sit (traction) reflex was on the borderline of statistical significance (*p* = 0.056) (Table 7).

The results of the analysis examining the relationship between the palmar grasp reflex and postural reactions in the CCD test are presented in Table 8. In females, the palmar grasp reflex was significantly correlated only with the pull-to-sit reflex and the Landau reaction (*p* = 0.006 and *p* = 0.035, respectively). In both genders, the palmar grasp reflex was not significantly correlated with the results of the remaining motor tests (Table 8).

## 4. Discussion

In the first year of life, SMA serves as a window into the development and functional state of the CNS. Neurodevelopmental disorders should be diagnosed with the use of standardized tools based on the results of thorough observations to identify potential correlations between a child’s spontaneous behavior and motor reflexes.

In the present study, an attempt was also made to analyze the relationships between vital signs at birth, the infants’ motor skills, IMPs, and CCDs. In physical therapy practice, the identified correlations could facilitate diagnosis and shorten the time from diagnosis to treatment. This study revealed significant correlations only for some vital signs at birth; in no case were they observed simultaneously in both genders. These findings suggest that the identified relationships may indicate a risk of neurodevelopmental disorders; however, they cannot serve as definitive predictors of such atypical conditions.

The analyzed relationships indicate that vital signs at birth, including birth weight and Apgar score, as well as the infant’s age during the examination, can be associated with developmental anomalies in females but do not play a key role because they proceed differently in both genders. In turn, SMA and IMPs are related to CCDs in male infants. The analyses that we conducted indicated that, in males, there was an interdependence between the CCD assessments and SMA, as well as between CCDs and IMPs, which appeared to be influenced by delayed neurological maturation. In female subjects, no such interdependence was observed. In females, CCDs did not significantly affect the development of spontaneous motor activity or movement patterns. Despite the presence of these disorders, the studied infants continued to acquire subsequent motor skills, although they did not always display the ideal movement patterns. Male and female subjects differed in baseline characteristics; therefore, it might be more informative to compare the deficits or gains in psychomotor skills relative to the infant’s gestational age rather than the raw data. This approach could provide a clearer picture of the differences in neurological maturation between the genders.

This observation suggests that newborns who have not achieved motor development milestones or whose motor skills deviate from the physiological standard should be diagnosed for CNS disorders and referred for neurodevelopmental therapy as soon as possible.

In the current study, the risk of CCD was not correlated with gestation length, birth weight, or Apgar score. The above could be attributed to the fact that these associations were explored across the entire study population without division into subgroups characterized by short (up to 37 weeks) and typical gestation lengths (40 weeks), low birth weight (up to 2500 g) and macrosomia (birth weight higher than 4000 g), or low Apgar scores (≤4 points). Therefore, further and more detailed research is needed to explore potential differences between these subgroups.

In many review and cohort studies conducted in Denmark, Italy, and Canada, perinatal parameters (such as birth weight, gestational age, and complications) had no significant impact on the development of CCDs/DCD [28,29,30]. A similar phenomenon was described in systematic reviews [31]; only in cases of extremely low birth weight, some factors (such as the male gender) had predictive value, but, overall, negative findings were reported in the literature [32]. Parameters such as birth weight and Apgar score assess perinatal conditions, which do not necessarily reflect the neurological status of the newborn. In contrast, the Vojta assessment is an independent diagnostic tool evaluating the state and maturity of the central nervous system. Furthermore, when assessing the neurophysiological development of preterm infants, the child’s chronological age is corrected for the number of weeks missing to reach full-term pregnancy. As a result, corrected age itself may not always serve as a predictive factor for the development of preterm infants.

Lipińska et al. [33] examined potential correlations between the progression of pregnancy and hyperactivity in children and adolescents aged 7–17 years. They found that the risk of neurodevelopmental disorders was higher in children who received an Apgar score of 0–4 points at 1 min and 5 min after birth. In turn, Kubisa [34] evaluated the psychomotor development of preterm infants and reported no significant correlations (*p* > 0.05) between the Apgar score at 1, 3, 5, and 10 min after birth and the psychomotor development of infants in the first year of life. The present study revealed a significant correlation between the Apgar score (*p* < 0.05) and the psychomotor abilities (SMA and IMPs) of females, but not males. These observations suggest that the Apgar score is not always a reliable predictor of typical or atypical psychomotor development; therefore, the decision to refer infants for motor skills therapy should not be based solely on this parameter.

In a cohort study examining infants aged up to 5 months, Kwong et al. [35] attempted to identify diagnostic tools for evaluating SMA and capturing the activity repertoire of newborns who were later diagnosed with cerebral palsy. The cited authors conducted a thorough analysis of the literature to identify studies investigating the SMA of infants using Prechtl’s General Movements Assessment. They concluded that the SMA and motor skills of infants are strong predictors of neurodevelopmental disorders and cerebral palsy. The cited authors also attempted to demonstrate that motor skills and IMPs are associated with CNS disorders in newborns. They found that male infants’ scores in the SMA and IMP tests deteriorated substantially with an increase in the number of atypical postural reactions in the CCD test. Espel et al. [36] conducted a cohort study of 232 mothers and their term newborns (50.4% males) who were assessed at three time points in the first postnatal year. An analysis of the relationship between gestation length and the child’s cognitive and motor development revealed that a longer pregnancy, even in children that were born at full term, promotes mental and motor development and that preterm infants show persisting impairments in cognitive function, school achievement, and brain development. In turn, Zachwieja [37] investigated the psychomotor development of 283 preterm children and found that the risk of pediatric cerebral palsy increases with shorter gestational length. The cited studies indicate that the length of pregnancy affects the development and potential anomalies of the CNS in newborns. This observation was not corroborated by the present study, which found no significant correlations between neurodevelopmental disorders and gestational length in male or female infants.

In this study, an attempt was also made to analyze the relationships between the Moro reflex, the palmar grasp reflex, and seven postural reactions. Age-appropriate involuntary reactions were evaluated in each of the seven tested postures. The assessment revealed a significant correlation between both involuntary reactions and the postural reaction. In females, the grasp reflex was significantly correlated with an atypical pull-to-sit reflex and an atypical Landau reaction. Prolonged retention of the grasp reflex is typically related to unusually high muscle tone in the upper limbs, which may explain the postural reactions observed in the study. In male infants, the Moro reflex was correlated with the Vojta reaction and the Collis vertical suspension response. In addition, females and males diagnosed with postural asymmetry were also characterized by higher CCD scores. The above suggests that postural asymmetry is significantly correlated with CCD and could be a predictor of atypical psychomotor development in later life.

Surowińska et al. [38] examined 107 newborns at 3 months of age, with follow-up checks at 9 and 16 months, and they found that qualitative assessments were better predictors of developmental milestones than any of the reflexes, excluding walking, which is strongly predicted by the Babkin reflex. Chinello et al. [39] observed that the persistence of primitive reflexes, including the sucking reflex and palmar and plantar grasp reflexes, can be promising early indicators of autism spectrum disorders in children. The clinical significance of palmar and plantar grasp reflexes was also demonstrated by Futagi et al. [40]. The cited researchers concluded that these reflexes are sensitive and clinically significant predictors of atypical neural development, particularly in children with spastic cerebral palsy.

Neurodevelopmental assessments of neonates should be conducted as early as possible, especially in children at risk of developmental anomalies. The risk factors that predispose infants to neurodevelopmental disorders include obstructed labor, maternal hygiene and lifestyle, maternal medical history, and maternal physical activity [41,42], which were not considered in the current study. These assessments can be conducted using numerous diagnostic tools that were discussed in the study. These tools should be carefully selected based on the examiner’s skills and experience, as well as the child’s age, to ensure that neonates’ psychomotor development is evaluated objectively and that the results of the assessment can be reliably used to choose the optimal treatment.

The clinical observations and scientific analyses presented in this publication clearly indicate that the persistence of infantile reflexes may serve as a predictive factor for atypical motor development. Confirmation of this diagnosis can be supported by assessment using the Vojta method, which is an effective tool for diagnosing and treating infants; however, its effectiveness depends on the timing of the intervention and the specificity of the underlying problems. In pediatric practice, it has been consistently observed that early identification and therapy with the Vojta method provide measurable developmental and psychosocial benefits with a long-term impact.

## 5. Strengths and Limitations

The strength of this study lies in the comprehensive analysis of the correlations between numerous variables, including separate assessments of perinatal factors in each gender. The principal limitation, however, is that the study population was not stratified into age subgroups defined by specific risk factors, such as preterm birth, low birth weight, or low Apgar scores. Additional age-based stratification would have substantially broadened the scope of the analysis, thus increasing the length of the manuscript. Furthermore, age-based stratification could lead to insufficient numbers of subjects within certain age categories, potentially necessitating the exclusion of newborns from specific groups, thereby reducing the overall sample size.

## 6. Conclusions

Vital signs at birth, including birth weight, Apgar score, and type of delivery, can be useful predictors of motor development disorders but do not clearly discriminate infants who require neurodevelopmental therapy. The grasp reflex, the Moro reflex, and postural asymmetry are significantly correlated with CCDs and could be indicative of potential irregularities in the psychomotor development of infants. Special attention should be paid to the prolonged retention of motor reflexes to diagnose neonates at risk of atypical psychomotor development and to initiate appropriate treatment as early as possible.

## Figures and Tables

**Table 1:** Characteristics of the studied male and female infants.

Variable	Male *n* = 107				Female *n* = 90				*t*	*p*
	Mean	SD	Min	Max	Mean	SD	Min	Max		
Gestation length [weeks]	37.5	3.5	24	42	38.4	2.7	26	42	−1.92	0.055
Birth weight [g]	3118.5	859.2	666	4900	3078.4	783.2	660	4450	0.34	0.734
Apgar score * [points]	8.9	2.0	1	10	9.3	1.43	2	10	−1.70	0.091
Age [months]	4.2	2.2	1	16	3.8	2.1	1	11	0.99	0.324

**Notes:** *—in the first minute after birth.

**Table 2:** Scores received by male and female infants in CCD, SMA, and IMP tests.

Variable	Male *n* = 107			Female *n* = 90			*t*	*p*	D-Cohen′s
	Mean	SD	Min–Max	Mean	SD	Min–Max			
CCDs	4.61	2.17	0–7	5.06	2.18	0–7	−1.44	0.151	0.202
SMA	3.42	2.02	0–11	2.74	2.07	0–9	2.31	**0.022**	**0.332**
IMPs	1.68	2.01	0–8	1.39	2.05	0–8	0.99	0.321	0.143

**Notes:** CCDs—central coordination disorders, SMA—spontaneous motor activity, IMPs—ideal movement patterns, bold font—statistically significant difference.

**Table 3:** Relationships between SMA and IMP scores and vital signs at birth in male and female infants.

Variable		Male (*n* = 107)		Female (*n* = 89)	
		*r*	*p*	*r*	*p*
SMA	Gestation length [weeks]	−0.06	ns	0.12	ns
	Birth weight [g]	−0.04	ns	**0.24**	**0.023**
	Apgar score [points]	−0.12	ns	**0.22**	**0.048**
	Age [months]	**0.71**	**<0.001**	**0.76**	**<0.001**
IMP	Gestation length [weeks]	0.07	ns	0.14	ns
	Birth weight [g]	0.06	ns	**0.27**	**0.011**
	Apgar score [points]	−0.02	ns	**0.24**	**0.025**
	Age [months]	**0.64**	**<0.001**	**0.75**	**<0.001**

**Notes:** ns—not significant, bold font—statistically significant correlation.

**Table 4:** Relationships between CCD scores and vital signs at birth in male and female infants.

Variable		Male (*n* = 107)		Female (*n* = 89)	
		*r*	*p*	*r*	*p*
CCD	Gestation length [weeks]	0.09	ns	−0.05	ns
	Birth weight [g]	0.04	ns	−0.11	ns
	Apgar score [points]	0.01	ns	−0.14	ns
	Age [months]	−0.09	ns	0.17	ns

**Notes:** ns—not significant.

**Table 5:** Relationships between the CCD score and SMA and IMP scores in male and female infants.

Variable		Male (*n* = 107)		Female (*n* = 89)	
		*r*	*p*	*r*	*p*
CCD	SMA	**−0.23**	**0.017**	−0.05	ns
	IMP	**−0.36**	**<0.001**	−0.11	ns

**Notes:** ns—not significant, bold font—statistically significant difference.

**Table 6:** Relationships between CCD scores, involuntary motor reflexes, postural asymmetry, and type of delivery in male and female infants.

Variable		Male				Female				Differences	
		*n*	Mean	SD	Min–Max	*n*	Mean	SD	Min–Max	*t*	*p*
Gender		107	4.61	2.17		90	5.06	2.18		1.44	ns
Type of delivery	Spontaneous	20	4.90	1.71	2–7	16	5.07	2.32	0–7	0.24	ns
	C-section	36	4.14	2.37	0–7	26	5.54	2.04	0–7	**2.43**	**0.018**
	*t* (*p*)	ns				ns					
Palmar grasp reflex	Absent	41	4.17	2.17	0–7	30	4.33	2.23	0–7	0.31	ns
	Present	52	4.81	2.11	0–7	46	5.34	2.16	0–7	1.25	ns
	*t* (*p*)	ns				**1.99 (0.049)**					
Moro reflex	Absent	44	4.30	2.18	0–7	28	5.14	2.12	0–7	1.62	ns
	Present	19	6.00	1.45	3–7	18	5.17	2.41	0–7	1.29	ns
	*t* (*p*)	**3.11 (0.003)**				ns					
Postural asymmetry	Absent	16	3.13	2.22	0–7	13	3.15	2.48	0–7	0.03	ns
	Present	88	4.87	2.06	0–7	73	5.45	1.95	0–7	1.78	ns
	*t* (*p*)	**3.12 (0.002)**				**3.75 (<0.001)**					

**Notes:** ns—not significant, bold font—statistically significant difference.

**Table 7:** Relationships between the Moro reflex and seven postural reactions in the CCD test.

Variable/Category		Male		Female	
		Present (*n* = 19)	Absent (*n* = 44)	Present (*n* = 18)	Absent (*n* = 28)
Pull-to-sit test		*χ^2^* = 1.28, *γ* = 0.42, *p* = 0.258		*χ*^2^ = 3.65, *γ* = 0.58, *p* = 0.056 *	
0	*fo*	2	10	7	4
	*fo–fe*	−1.62	1.62	2.70	−2.70
1	*Fo*	17	34	11	24
	*fo–fe*	1.62	−1.62	−2.70	2.70
Landau reaction		*χ*^2^ = 3.74, *γ* = 0.63, *p* = 0.053 *		*χ*^2^ = 0.01, *γ* = 0.04, *p* = 0.917	
0	*fo*	2	15	3	5
	*fo–fe*	−3.13	3.13	−0.13	0.13
1	*fo*	17	29	15	23
	*fo–fe*	3.13	−3.13	0.13	−0.13
Auxiliary suspension test		*χ*^2^ = 0.75, *γ* = 0.27, *p* = 0.385		*χ*^2^ = 1.96, *γ* = 0.47, *p* = 0.161	
0	*fo*	4	14	3	10
	*fo–fe*	−1.43	1.43	−2.09	2.09
1	*Fo*	15	30	15	18
	*fo–fe*	1.43	−1.43	2.09	−2.09
Vojta reaction		***χ*** **^2^ = 6.37, *γ* = 0.73, *p* = 0.012**		*χ*^2^ = 0.01, *γ* = 0.02, *p* = 0.953	
0	*fo*	2	19	5	8
	*fo–fe*	−4.33	4.33	−0.09	0.09
1	*fo*	17	25	13	20
	*fo–fe*	4.33	−4.33	0.09	−0.09
Collis horizontal suspension test		*χ*^2^ = 2.81, *γ* = 0.44, *p* = 0.094		*χ*^2^ = 0.12, *γ* = 0.11, *p* = 0.732	
0	*Fo*	6	24	6	8
	*fo–fe*	−3.05	3.05	0.52	−0.52
1	*fo*	13	20	12	20
	*fo–fe*	3.05	−3.05	−0.52	0.52
Peiper-Isbert suspension test		*χ*^2^ = 3.18, *γ* = 0.60, *p* = 0.074		*χ*^2^ = 0.23, *γ* = 0.17, *p* = 0.632	
0	*fo*	2	14	4	8
	*fo–fe*	−2.83	2.83	−0.70	0.70
1	*fo*	17	30	14	20
	*fo–fe*	2.83	−2.83	0.70	−0.70
Collis vertical suspension test		***χ*** **^2^ = 12.43, *γ* = 0.90, *p* < 0.001**		*χ*^2^ = 0.10, *γ* = 0.10, *p* = 0.754	
0	*fo*	1	23	5	9
	*fo–fe*	−6.24	6.24	−0.48	0.48
1	*fo*	18	21	13	19
	*fo–fe*	6.24	−6.24	0.48	−0.48

**Notes:** * Borderline significant; *fo*—frequency observed; *fe*—frequency expected, ns—not significant, bold font—statistically significant relationship.

**Table 8:** Relationships between the palmar grasp reflex and seven postural reactions in the CCD test.

Variable/Category		Male		Female	
		Present (*n* = 52)	Absent (*n* = 41)	Present (*n* = 46)	Absent (*n* = 30)
Pull-to-sit test		*χ*^2^ = 2.63, *γ* = 0.38, *p* = 0.105		***χ*** **^2^ = 7.57, *γ* = 0.61, *p* = 0.006**	
0	*fo* *fo-fe*	9	13	8	14
		−3.30	3.30	−5.32	5.32
1	*fo*	43	28	38	16
	*fo–fe*	3.30	−3.30	5.32	−5.32
Landau reaction		*χ*^2^ = 2.71, *γ* = 0.37, *p* = 0.010		***χ*** **^2^ = 4.42, *γ* = 0.56, *p* = 0.035**	
0	*fo*	11	15	5	9
	*fo–fe*	−3.54	3.54	−3.47	3.47
1	*fo*	41	26	41	21
	*fo–fe*	3.54	−3.54	3.47	−3.47
Auxiliary suspension test		*χ*^2^ = 0.93, *γ* = 0.22, *p* = 0.335		*χ*^2^ = 1.09, *γ* = 0.28, *p* = 0.296	
0	*fo*	13	14	9	9
	*fo–fe*	−2.10	2.10	−1.89	1.89
1	*fo*	39	27	37	21
	*fo–fe*	2.10	−2.10	1.89	−1.89
Vojta reaction		*χ*^2^ = 2.37, *γ* = 0.32, *p* = 0.124		*χ*^2^ = 2.69, *γ* = 0.38, *p* = 0.101	
0	*fo*	16	19	13	14
	*fo–fe*	−3.57	3.57	−3.34	3.34
1	*fo*	36	22	33	16
	*fo–fe*	3.57	−3.57	3.34	−3.34
Collis horizontal suspension test		*χ*^2^ = 0.05, *γ* = 0.05, *p* = 0.829		*χ*^2^ = 0.13, *γ* = 0.09, *p* = 0.715	
0	*fo*	28	23	15	11
	*fo–fe*	−0.52	0.52	−0.74	0.74
1	*fo*	24	18	31	19
	*fo–fe*	0.52	−0.52	0.74	−0.74
Peiper-Isbert suspension test		*χ*^2^ = 1.14, *γ* = 0.24, *p* = 0.286		*χ*^2^ = 0.96, *γ* = 0.24, *p* = 0.326	
0	*fo*	18	10	12	11
	*fo–fe*	2.34	−2.34	−1.92	1.92
1	*fo*	34	31	34	19
	*fo–fe*	−2.34	2.34	1.92	−1.92
Collis vertical suspension test		*χ*^2^ = 2.73, *γ* = 0.34, *p =* 0.099		*χ*^2^ = 0.74, *γ* = 0.21, *p* = 0.390	
0	*fo*	19	22	14	12
	*fo–fe*	−3.92	3.92	−1.74	1.74
1	*fo*	33	19	32	18
	*fo–fe*	3.92	−3.92	1.74	−1.74

**Notes:** *fo*—frequency observed, *fe*—frequency expected, ns—not significant, bold font—statistically significant relationship.

## Data Availability

The access to Excel data generated during this study has been restricted by the Ethics Committee of the UWM in Olsztyn to protect the participants’ privacy. Researchers who meet the criteria for access to confidential data can submit a data request by email to podstawskirobert@gmail.com.

## Abbreviations

The following abbreviations are used in this manuscript:

SMA	spontaneous motor activity
IMPs	ideal movement patterns
CCDs	central coordination disorders
MRI	magnetic resonance imaging
